# Screening and brief intervention delivered simultaneously in multiple settings: it is cost-effective, but can it influence community-level outcomes?

**DOI:** 10.1186/1940-0640-7-S1-A13

**Published:** 2012-10-09

**Authors:** Anthony Shakeshaft

**Affiliations:** 1 National Drug and Alcohol Research Center, University of New South Wales, Sydney, Australia

## 

A 20-community randomized controlled trial, the Alcohol Action in Rural Communities (AARC) project, provided the opportunity to examine the cost-effectiveness of screening and brief intervention (SBI) delivered simultaneously in general practice (GP), pharmacy, and emergency department (ED) settings and the community level impact of the SBI on problem drinking. For the GP- and pharmacy-delivered SBI, decision models and scenario analysis assessed outcomes and costs in the 10 experimental communities of the trial. For the ED-delivered SBI, a randomized controlled trial design was used to examine the cost-effectiveness of mailed personalized feedback. For both the GP- and pharmacy-delivered SBI, the most cost-effective outcome was to increase screening alone: GPs and pharmacies screening all patients achieved an incremental cost-effectiveness ratio (ICER) of AUD $197 and AUD $29, respectively, per risky drinker who reduced drinking. The ED-based SBI resulted in a reduction of 2.6 fewer drinks per week at an average cost of $5.55 per patient and an ICER of $2.13 per one standard drink reduction in average weekly consumption. In addition to cost-effectiveness, the AARC community approach provided the opportunity to analyze the effect of SBI on community level outcomes. Currently, 19% of risky drinkers in a community visit a GP and reduce their drinking, which would increase to 36% if all patients got SBI. Similarly, 23% of risky drinkers in a community visit a pharmacy and reduce their drinking, which would increase to 34% if they all got SBI. Although our results confirm SBI is cost-effective, the impact at the community level is unclear: if all GPs and pharmacists delivered SBI to all their risky drinking patients, only 34-36% would reduce their drinking. A trial that assessed the impact of SBI delivered in multiple settings simultaneously on community level indicators of alcohol harm would move the field toward demonstrating the cost benefit, as well as cost-effectiveness, of SBI.

